# Evaluation of anti-malarial potency of new pyrazole-hydrazine coupled to Schiff base derivatives

**DOI:** 10.1186/s12936-022-04266-8

**Published:** 2022-08-22

**Authors:** Akachukwu Ibezim, Martha N. Ofokansi, Xavier Ndukwe, Chidera S. Chiama, Bonaventure C. Obi, Ogechukwu N. Isiogugu, Peter E. Ikechukwu, Akachukwu M. Onwuka, Stella A. Ihim, Jonnie N. Asegbeloyin, Ngozi J. Nwodo

**Affiliations:** 1 Department of Pharmaceutical and Medicinal Chemistry, University of Nigeria, Nsukka, Nigeria; 2 Department of Pharmacology and Toxicology, University of Nigeria, Nsukka, Nigeria; 3 Department of Pure and Industrial Chemistry, University of Nigeria, Nsukka, Nigeria; 4 Department of Science Laboratory, University of Nigeria, Nsukka, Nigeria

**Keywords:** Antimalarial, Pyrazole, Hydrazine, Schiff-base, Hematology, Docking

## Abstract

**Background:**

The search for pharmacologically effective agents among molecules bearing multiple functionalities is commonly practiced. In continuation of the search for new anti-malarial agents, new pyrazole-hydrazine coupled Schiff-base derivatives previously synthesized were screened for anti-malarial property.

**Methods:**

Here, in vivo prophylactic and curative activities of the compounds were assessed while their binding affinity for falcipain-2, a crucial enzyme in *Plasmodium* survival, was done using computational techniques.

**Results:**

The two derivatives (BepINH and BepBeH) respectively led to a significant (*p* < 0.05) reduction in parasitaemia count (0.76 ± 1.11 and 0.79 ± 1.19) at day 3 post-treatment relative to the negative control (16.37 ± 1.25). For the prophylactic study, it was observed that the highest parasitaemia suppression level of 95.35% and 95.17% for BepINH and BepBeH at 15 mg/kg was slightly comparable to that obtained for ACT-Lonart (99.38%). In addition, their haematological profiles indicate that they are potentially beneficial in suppressing haemolytic damage to RBC, thereby protecting the body against infection-induced anaemia. Docking calculations on the derivatives toward the *Plasmodium falciparum* falcipain-2 revealed that they favourably interacted with a binding affinity higher than that of a known cocrystallized inhibitor.

**Conclusion:**

This study confirms the relevance of multi-functional molecules in the search for new and effective anti-plasmodial agent and lay the foundation for further development of these compound series to potent anti-plasmodial agent that interacts with falcipain-2.

**Supplementary Information:**

The online version contains supplementary material available at 10.1186/s12936-022-04266-8.

## Background

Malaria is a serious health burden with a significant impact on morbidity and mortality worldwide. Recent estimates by the World Health Organization (WHO) indicate that there were about 229 million new cases of malaria in 2019, leading to 409,000 malaria deaths. On average, 60–90% of malaria cases are in the African region compared to 7% in the Southeast Asia region and 2% in the Eastern Mediterranean region [1]. It is caused by infection of red blood cells with the parasites of the genus *Plasmodium*. When ineffective or poor-quality medication is used, particularly in the case of *Plasmodium falciparum* malaria, the parasite burden often continues to increase and the patient may develop potentially lethal severe malaria [2, 3]. Despite remarkable progress in combating the disease, the prevalence of drug-resistant parasites, non-compliance to treatment schemes and drug counterfeiting continue to pose a major challenge to achieving global malaria control [4, 5]. During the morphologically separate phases inside the red blood cell, the malaria parasites degrade host cell haemoglobin for food and to create space for their growth in the erythrocyte. Interception of this process results to the death of the parasite. At the early trophozoite stage, falcipain-2 is used to catabolize haemoglobin and to destabilize the red blood cell membrane at the schizont stage. Hence, it is a vital target for discovering anti-malarial candidates [6–10].

This work is based on the hypothesis that molecules bearing multiple functionalities demonstrate excellent biological activity [11, 12]. Hence, studies were carried out on the anti-malarial effects of pyrazole-hydrazine coupled to Schiff base derivatives (PHCSB), which comprises three functionalities: pyrazole, hydrazine and Schiff-base, earlier reported [13–15]. Moreover, many studies have employed this strategy in search of novel anti-malarial agents. For example, Vandekerckhove and D’hooghe [16] reviewed several works on anti-malarial that used hybrid molecules containing a quinoline scaffold, while Aggarwal and colleagues revealed two pyrazole Schiff-based hybrids that showed EC_50_ 1.95 µg/ml and 1.98 µg/ml against *P. falciparum* asexual blood stages [17].

## Methods

### Biological screening

#### Acute toxicity (LD_50_) study

An acute toxicity study of PHCSBD was carried out using Lorke’s method [18], with slight modifications. Briefly, varying doses of PHCSBD (2.5 mg/kg, 10 mg/kg, 20 mg/kg, 40 mg/kg, 100 mg/kg) were orally administered to five groups of mice (n = 3) and observed strictly for clinical signs and symptoms of toxicity in the first phase of the study. In the second phase, the PHCSBD at higher doses (200 mg/kg, 250 mg/kg and 300 mg) were administered. In each phase, the animals were observed for signs of toxicity and/or mortality for 24 h. The (LD50) of PHCSBD was calculated as the geometric mean of the lowest lethal dose and highest non-lethal dose.

#### Anti-plasmodial study

Adult Swiss albino mice (18–25 g), obtained from the Animal House Facility of the Department of Pharmacology and Toxicology, University of Nigeria, Nsukka, were used for the study. The animals were housed at 25 ± 2 °C under a 12-h light/dark cycle maintained on standard pellets (Guinea Feed Nigeria, Ltd.) with free access to water ad libitum. The animals were allowed to acclimatize for 7 days prior to the study. Each mouse was inoculated with 0.6 ml of infected blood containing 1 × 10^7^ parasite cells of *Plasmodium* from a donor mouse. Parasitaemia level was determined microscopically (400 × magnification) by counting the average number of parasites in 10 fields of at least 1000 erythrocytes. All animal experiments were conducted in compliance with the National (US) Institute of Health Guide for Care and Use of Laboratory Animals (Pub No. 85–23, revised 1985) and in accordance with the University of Nigeria Ethics Committee on the use of laboratory animals, registered by the National Health Research Ethics Committee (NHREC) of Nigeria.

#### Prophylactic test

The prophylactic activity of PHCSBD was carried out against *P. berghei* (NK-65 strain) infected mice as described by Fidock et al. [19], with slight modifications. Forty mice (n = 5) placed in five (5) groups were administered different doses of PHCSBD and the standard drug for 4 consecutive days as stated below:

Group A: control (untreated);

Group B: received 2.5 mg/kg/BePINH cpd.

Group C: received 10 mg/kg/BePINH cpd.

Group D: received 15 mg/kg/ BePINH cpd.

Group E: received 2.5 mg/kg/BePBeH cpd.

Group F: received 10 mg/kg/BePBeH cpd.

Group G: received 15 mg/kg/BePBeH cpd.

Group H: received 5 mg/kg Artemether Combination Therapy (ACT-Lonart).

BePINH: (Z)-3-Methyl-1-phenyl-4-(2-phenylhydrazono)-1H-pyrazol-5(4H)-one.

BePBeH: (E)-4-((2-benzylhydrazono) (phenyl)methyl)-3-methyl-1-phenyl-1H-pyrazol-5-ol.

Twenty-four hours after the last administration (day 0), the mice were all inoculated intraperitoneally with 0.2 ml of blood containing 1 × 10^7^ cells/ml. Parasitaemia counts were estimated 72 h (day 3) after inoculation and subsequently on the 4th and 5th day post-inoculation. The percentage suppression in parasitaemia count was calculated using the formula:

$${\text{PPS }} = {\text{ }}\left[ {\left( {{\text{A}} - {\text{B}}} \right)/{\text{A}}} \right]{\text{ }} \times {\text{ 1}}00$$

where PPS = percentage parasitaemia suppression; A = mean parasitaemia count of control (untreated)/zero hour/days; B = mean parasitaemia count of treated groups/given hour/days.

#### Curative test

Evaluation of the schizontocidal activity of PHCSBD compared to artemisinin-based combination therapy (artemether/lumefantrine) against *P. berghei* infection in mice was carried out using a 4-day suppressive test [20], with slight modifications. Forty mice (n = 5) divided into five (5) groups were infected with 0.2 ml of blood containing 1 × 10^7^ parasitized red blood cells on day 0. Seventy-two hours later, after confirmation of parasitaemia (Day 0), group 1 (untreated control) received 0.2 ml distilled water, groups 2, 3, 4 received BePINH at 2.5, 10 and 15 mg/kg; groups 5, 6, 7 received BePBeH at 2.5, 10 and 15 mg/kg; group 8 received the standard drug (artemether-lumenfantrine) at 5 mg/kg. Treatment was continued daily at the same time of the day for 4 consecutive days intraperitoneally. Subsequently, parasitaemia counts were monitored on each day of the treatment. The number of parasitized cells and the percentage of parasitaemia suppression were determined as described above.

Blood samples were collected from the animals via ocular puncture for analysis of haematological parameters, such as red blood cell count (RBC), white blood cell count (WBC), packed cell volume (PCV), and haemoglobin (Hb) concentrations, using standard methods [21].

#### Molecular modelling

The coordinate of *P. falciparum* falcipain-2 (FP2) along with its cocrystallized inhibitor (E64) were retrieved from the protein databank (pdb code 3BPF) [22] and the complex was prepared for molecular simulation purposes following standard procedures described [23, 24]. Water and non-essential small molecules were deleted and hydrogen atoms were added to the FP2-E64 complex with Protonate 3D Wizard of Molecular Operating Environment (MOE) (Molecular Operating Environment, version 2014). The complex was energy minimized to a gradient of 10–5 kcal/mol with Merck Molecular (MMFF94) forcefield [25] to relax atom coordinates and prevent atomic clashes. Finally, E64 was detached from the FP2 binding site and both molecules were saved separately as a.pdb file.

The molecular builder interface implemented in MOE was used to generate the three-dimensional structures of the PHCSBD, energy minimized to the above gradient and saved as a.pdb file. Two online-based programs designated as Molinspiration [26] and pkCSM [27] were used to compute some molecular descriptors presented in the result and discussion section.

AutoDock 4.2 software was used to predict the binding poses of the PHCSBD in the FP2 binding site and their theoretical binding free energy. In preparing the molecules for docking, the AutoDock Tools program [28] was used to add atomic Gasteiger partial charges to the ligands and set its active torsion to a maximum of 6 and calculate the potential interaction grid map for the FP2 structure. Prior to calculations of the potential maps for the interaction of ligand atom-types by the AutoGrid program, a grid box covering FP2 active site residues, measured within 5.0 Å from the cocrystallized ligand, was centered in the mass centre of E64. During docking calculations, 250 hybrid Genetic Algorithm (GA) runs were carried out for a maximum of 2.5 M energy evaluations and a maximum of 27 000 generations. Except for a root-mean-square deviation (rmsd) tolerance of 2.0 Å set for cluster grouping and the degree of freedom and ligand coordinates set at random, the rest of the AutoDock parameters were kept at default during the rigid protein-flexible ligand docking calculations. AutoDock 4.2 evaluates the dock conformations using a semi-empirical free energy force field which computes a number of energies such as final intermolecular energy (comprising van dar Waal, hydrogen bond, desolvation and electrostatic energies), final total internal energy, torsional free energy and unbound system’s energy, to arrive at the estimated free energy of binding (kcal/mol) and estimated inhibition constant (molar) of the docked ligand.

## Results and discussion

The emergence of drug-resistant strains of *Plasmodium* spp. is believed to be largely responsible for continued menace of malaria across a substantial number of the world population. Besides the use of more than one agent as a recommended method of handling treatment failures, the use of single molecules bearing more than one structurally active moieties is also considered a likely effective option. In view of that, here compounds comprising three different functionalities; pyrazole, hydrazine and Schiff-base, were evaluated for their anti-malarial activity.

### Physical features of the PHCSBD

To avert wasting resources on a molecule with a pharmacokinetic challenge, this property is determined from the start in drug development [29]. Sequel to that, some molecular descriptors of PHCSBD used to assess physicochemical properties of compounds were calculated using two online programs [26] and pkCSM [27] and their results were compared with values reported for the same parameters for 95% of known drugs. Given the recommended ranges for 95% of drugs, the two study compounds qualify as potential drug candidates, which would pose no metabolic and pharmacokinetic problems (Table 1). Additionally, regarding Lipinski’s rule of five (MW < 500, logP < 5, HBD ≤ 5 and HBA ≤ 10) [30], which generally guides the selection of orally bioavailable molecules, the PHCSBD are drug-like. This implied that the compounds can easily penetrate the cell membranes and be available in sufficient quantity to interact with receptors.

**Table 1:** Theoretical molecular descriptor of the PHCSBD

Compound	NRB	HBA	HBD	logP	TPSA	MW	Vol	BIP_caco-2_	logB/B	logS
BepBeH	5	6	2	4.01	79.51	396.45	358.70	1.39	−0.063	−4.07
BepINH	5	7	2	3.46	92.41	397.44	354.55	1.08	0.257	−0.44

Notice the difference in the physicochemical properties of BepBeH and BepINH due to the presence of nitrogen atom in the pseudo-benzaldehyde ring. This converted the hydrophobic phenyl group to hydrophilic pyridinyl group, thereby highlighting the relevance of chemical structure and their modifications to parameters that define physical features since replacing carbon atom with nitrogen atom significantly improved the permeability of BepINH across the blood–brain barrier, aqueous solubility and total polar surface area (Table 1).

Recommended ranges of molecular descriptors for 95% of known drugs are thus: NRB = number of rotatable bond (accepted range for 95% is 0–15), HBA = hydrogen bond acceptor (accepted range for 95% of drugs is 2–20), HBD = hydrogen bond donor (accepted range for 95% of drug is 0–6), logP = logarithm of partitioning coefficient between n-octanol and water phases (accepted range for 95% of drugs is −2 to 6), TPSA = total polar surface area (accepted range for 95% of drug is  < 140 Å2), MW = molecular weight (accepted range for 95% of drugs is 130–725 Da), Vol. = total volume of molecule enclosed by solvent-accessible molecular surface, in Å3 (probe radius 1.4 Å) (accepted range for 95% of drugs: 500–2000 Å3), BIP_caco-2_ = the predicted apparent Caco-2 cell membrane permeability, in Boehringer-Ingelheim scale (accepted range foe 95% of drugs is  < 5 low,  > 100 high in nm s−1), logB/B = the logarithm of predicted blood/brain barrier partition coefficient (accepted range for 95% of drugs is −3.0 to 1.0), logS = the logarithm of aqueous solubility (accepted range for 95% of drugs is −6.0 to 0.5).

### Experimental results

In the biological screening test, schizonticidal activity of the PHCSBD was compared to artemisinin-based combination therapy (ACT: Lonart DS containing 80 mg/480 mg of artemether and lumefantrine, respectively) in *P. berghei* infected rodent model. In the acute toxicity test, the result showed that the PHCSBD did not produce any obvious signs of toxicity or mortality up to the highest dose of 100 mg/kg in the first phase of the study. In contrast, the PHCSBD at higher doses (200 mg/kg, 250 mg/kg and 300 mg) caused 100% death in the second phase of the study. The LD50 is the geometric mean of the highest non-lethal dose and the lowest lethal dose, thus:

$${\text{LD}}_{{{\text{5}}0}} = {\text{ }}(\sqrt {100} \times 200){\text{ }} = {\text{141}}.{\text{42}}$$

**Fig. 1:**
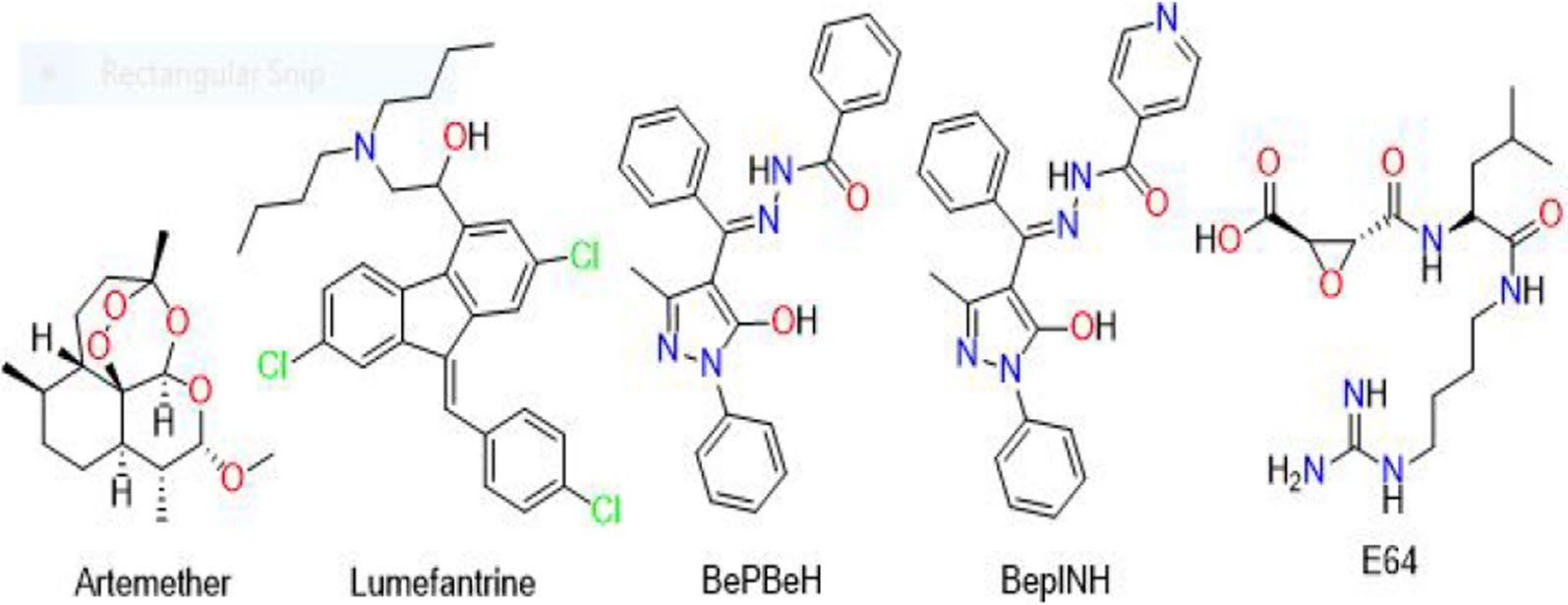
2-dimensional chemical structures of the compounds used in this study

Based on that, the compounds were judged to have a moderate safety profile and subsequent studies were conducted using concentrations quite below 100 mg/kg since a narrow therapeutic index does not deter the pharmacological activity and therapeutic usefulness within the limits of the therapeutic index of a potential drug candidate. Figure 1 shows the chemical structures of artemether and lumefantrine (ACT Lonart), BePBeH and BePINH (PHCSB derivatives).

The changes in parasitaemia induced by the inoculation of *P. berghei* are consistent with the results of previous investigators [31, 32]. As presented in Table 2 and Additional file 1: Figure S1, evident increases (*p* < 0.05) in percentage parasitaemia in control (untreated inoculated mice) were noticed on day 4 (14.40 ± 0.45) and day 5 (16.37 ± 1.25) post-inoculation compared with 9.40 ± 1.23 for day 3. Interestingly, in the prophylactic study, the PHCSBD showed a protective ability against *P. berghei* infection, indicating a promising therapeutic potential against malaria. Treatment with BePINH evoked significant (*p* < 0.05) reduction in parasitaemia count by (2.92 ± 1.64, 2.77 ± 1.65) 2.5 mg/kg, (2.30 ± 2.72, 2.05 ± 0.72) 10 mg/kg and (0.83 ± 0.92, 0.76 ± 1.11) 15 mg/kg at days 2 and 3, respectively when compared to day 1 post inoculation. Similarly, the BePBeH showed significant (*p* < 0.05) reduction in parasitaemia by (2.95 ± 0.49, 2.93 ± 1.06) 2.5 mg/kg, (2.50 ± 0.52, 2.18 ± 0.77) 10 mg/kg and (0.91 ± 1.62, 0.79 ± 1.19) 15 mg/kg at days 2 and 3, respectively. The standard drug (ACT-Lonart) elicited better activity in parasitaemia reduction by (0.26 ± 0.42, 0.10 ± 0.66). Suffice to say, the highest parasitaemia suppression level of 95.35% and 95.17% for A and B (at 15 mg/kg) was slightly comparable to that obtained for ACT-Lonart (99.38%), which indicate a better prophylactic effect at the highest doses.

**Table 2:** Prophylactic Study

Treatment	Dose (mg/kg)	Parasitaemia count		
		Day 3	Day 4	Day 5 (post inoculation)
Control	–	9.40 ± 1.23	14.40 ± 0.45^#^	16.37 ± 1.25^#^
BePINH	2.5	3.75 ± 1.08^*^	2.92 ± 1.64^*^	2.77 ± 1.65^*^
	10	3.70 ± 0.64^*^	2.30 ± 2.72^*^	2.05 ± 0.72^*^
	15	2.43 ± 0.86^*^	0.83 ± 0.92^*^	0.76 ± 1.11^*^
BePBeH	2.5	3.99 ± 0.74^*^	2.95 ± 0.49^*^	2.93 ± 1.06^*^
	10	3.72 ± 0.64^*^	2.50 ± 0.52^*^	2.18 ± 0.77^*^
	15	2.46 ± 0.75^*^	0.91 ± 1.62^*^	0.79 ± 1.19^*^
ACT	5	0.51 ± 0.26^*^	0.26 ± 0.42^*^	0.10 ± 0.66^*^

Each value represents the mean ± S.E.M, n = 5

*ACT* artemisinin-based combination therapy

^*^^,#^*p* < 0.05 compared with control and Day 3, respectively (One-way ANOVA; Dunnett’s post hoc)

In Table 3 and Additional file 1: Figure S2, similar plasmodial sensitivity was recorded. Significant (*p* < 0.05) increase in parasitaemia count was observed in the control group on day 1, which continued until day 3 (13.60 ± 0.62, 14.39 ± 0.27, 15.02 ± 0.53, respectively) when compared to (6.76 ± 0.29) at day 0. This observation is understandably justified as a major feature in *P. berghei* inoculated mice. In contrast, BePINH treated groups (2.5, 10, 15 mg/kg) elicited dose-dependent reductions (*p* < 0.05) in parasitaemia (7.53 ± 2.49, 6.56 ± 2.35, 6.28 ± 1.85) at day 1, which was sustained (6.61 ± 1.63, 5.58 ± 1.79, 5.53 ± 1.63) at day 2 and (6.33 ± 2.80, 5.04 ± 2.35, 2.93 ± 1.73) at day 3, respectively when compared to 7.70 ± 2.76, 8.26 ± 2.40 and 6.63 ± 2.02, respectively at day 0. Similarly, significant (*p* < 0.05) parasitaemia reduction was recorded for BePBeH treated groups (2.5, 10, 15 mg/kg) at day 1 (7.79 ± 2.06, 6.77 ± 1.92, 6.69 ± 1.77), day 2 (7.07 ± 1.65, 6.56 ± 2.11, 5.58 ± 1.55) and day 3 (6.64 ± 2.45, 5.31 ± 1.01, 2.98 ± 0.57), respectively. It is clear from the results that the suppressive ability of the PHCSBD is directly proportional to the dose used since for the different test groups, the percentage parasitaemia suppression increased with increasing doses. 15 mg/kg of both BePINH (80.49%) and BePBeH (80.16%) gave the highest inhibition. Based on this, it can be concluded that the two derivatives demonstrated interesting activity against malaria-causing pathogen and by extension, validates the relevance of molecules bearing multiple functionalities in malaria drug development.

**Table 3:** Curative study

Treatment	Dose (mg/kg)	Parasitaemia count			
		Day 0	Day 1	Day 2	Day 3
Control	–	6.76 ± 0.29	13.60 ± 0.62^#^	14.39 ± 0.27^#^	15.02 ± 0.53^#^
BePINH	2.5	7.70 ± 2.76	7.53 ± 2.49^*^	6.61 ± 1.63^*^	6.33 ± 2.80^*^
	10	8.26 ± 2.40	6.56 ± 2.35^*^	5.58 ± 1.79^*^	5.04 ± 2.35^*^
	15	6.63 ± 2.02	6.28 ± 1.85^*^	5.53 ± 1.63^*^	2.93 ± 1.73^*^
BePBeH	2.5	7.83 ± 2.58	7.79 ± 2.06	7.07 ± 1.65^*^	6.64 ± 2.45^*^
	10	6.63 ± 2.10	6.77 ± 1.92^*^	6.56 ± 2.11^*^	5.31 ± 1.01^*^
	15	6.35 ± 2.02	6.69 ± 1.77^*^	5.58 ± 1.55^*^	2.98 ± 0.57^*^
ACT	5	7.44 ± 2.01	6.66 ± 2.05^*^	3.03 ± 2.22^*^	1.40 ± 1.93^*#^

Each value represents the mean ± S.E.M, n = 5

*ACT* artemisinin-based Combination Therapy

^*^^,#^*p* < 0.05 compared with control and Day 0, respectively (One-way ANOVA; Dunnett’s post hoc)

The observation in haematological parameters is quite worthy as it corresponds with physiological changes during malarial infection [33]. Significantly (*p* < 0.05) lower amounts of the red blood cell count (RBC), white blood cell count (WBC), haemoglobin (Hb) and packed cell volume (PCV) were observed in the control group (Table 4) when compared to the test groups. Changes in the haematological profile are a consistent feature in malaria parasitaemia and represent a basic malaria infection pattern. In the control group, the observed decrease in blood parameters corroborates with previous reports documenting haematological indices of malaria*-*infected patients [34, 35] and increased RBCs breakdowns in *P. berghei-*infected mice*,* thus resulting in anaemia [32]. In marked contrast to the control, amounts of WBC, RBC, Hb and PCV in PHCSBD-treated groups were significantly (*p* < 0.05) higher (Table 4). Thus, in maintaining a normal physiologic level of RBC, WBC, Hb and PCV, the PHCSBD may be considered highly beneficial in suppressing haemolytic damage to RBC, thereby defending the body against infectious diseases and foreign substances.

**Table 4:** Effect of PHCSBD on haematological parameters

Treatment	Dose (mg/kg)	Parasitaemia count			
		RBC (10^6^/μL)	WBC (10^3^/μL)	PCV (%)	HB (g/dl)
Control	–	5.16 ± 3.88	8.22 ± 1..76	19.50 ± 1.59	5.75 ± 0.96
BePINH	2.5	9.81 ± 1.96	9.77 ± 1.37	38.25 ± 0.83^*^	10.63 ± 0.19^*^
	10	10.63 ± 1.28^*^	10.57 ± 2.02	36.50 ± 1.10^*^	11.03 ± 0.39^*^
	15	10.86 ± 1.11^*^	10.92 ± 1.70	37.25 ± 0.96^*^	12.18 ± 0.19^*^
BePBeH	2.5	8.24 ± 2.92	9.55 ± 1.56	36.87 ± 1.21^*^	10.71 ± 0.74^*^
	10	9.89 ± 1.31	10.41 ± 1.24	37.02 ± 0.92^*^	10.87 ± 0.81^*^
	15	10.31 ± 0.29^*^	10.85 ± 0.82	37.53 ± 1.01^*^	11.17 ± 1.25^*^
ACT	5	10.99 ± 1.17^*^	10.60 ± 1.70	41.50 ± 1.70^*^	12.00 ± 0.32^*^

Each value represents the mean ± S.E.M, n = 5

*ACT* Artemisinin-based Combination Therapy, *RBC* red blood cells, *WBC* white blood cells, *PCV* packed cell volume, *HB* haemoglobin

^*^p < 0.05 compared with control (One-way ANOVA; Dunnett’s post hoc)

### Docking results for the PHCSBD

Given the importance of haemoglobin degradation to *P. falciparum* erythrocytic survival, the ability of the PHCSBD to interrupt this process was investigated by considering falcipain-2 (FP2). FP2 is an important member of the cysteine proteases since it cleaves two cytoskeletal elements, ankyrin and protein 4.1, that are essential to the stability of red cell membranes at the schizont stage and its inhibition results to significant reduction in the hydrolysis of haemoglobin by trophozoites [9].

Before the docking study, the docking program has to be validated. In this study, the ability of the AutoDock Suite to reproduce the X-ray crystallographic conformation of the cocrystallized ligand to evaluate its reliability. In general, a dock program that retrieves a cocrystallized ligand dock poses comparable to the experimental pose by  ≤ 2.0 Å is acceptable [29]. Based on that, out of several grid parameters which were tried, the grid box of 40 X 40 X 40 points with a 0.375 Å point spacing centered on the mass centre (−57.837, −1.944, −15.427) of the crystallographic macromolecule encompassing all active site residues, gave a docking pose with rmsd of 0.96 Å from the experimental pose and hence was used to carry out docking of the PHCSBD.

**Fig. 2:**
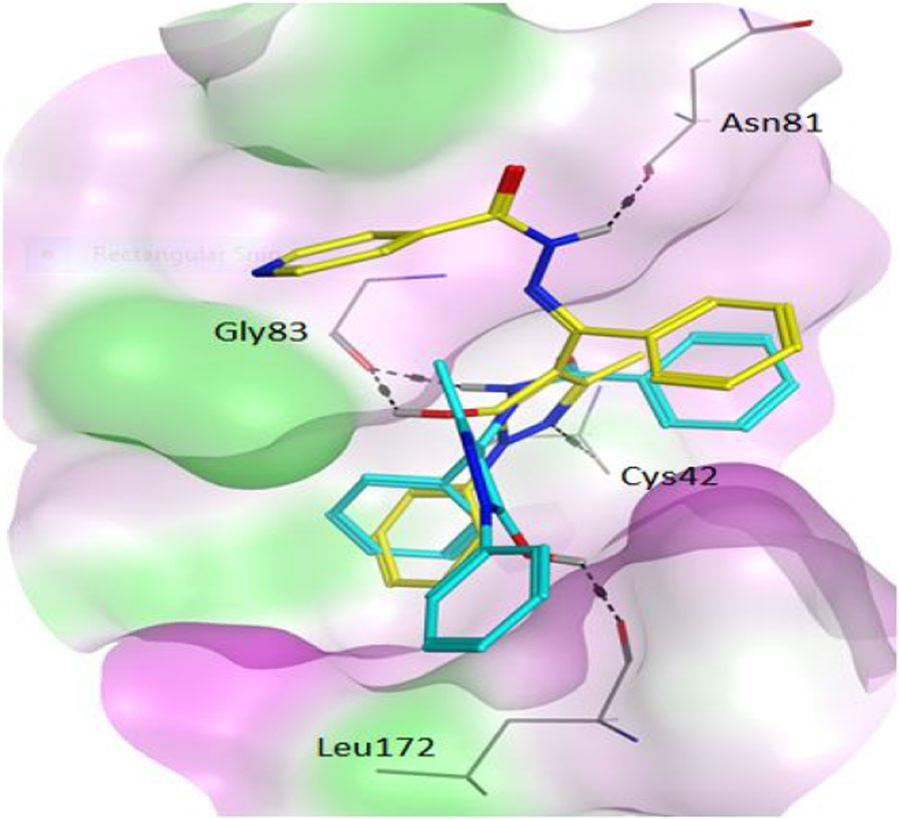
Theoretical binding poses of the pyrazole-hydrazine coupled schiff base derivatives (PHCSB) in FP2 active site. The surface map in which hydrophilic, lipophilic and neutral regions are respectively presented in purple, green and white colours while BepBeH and BepINH are shown in cyan and yellow colours, respectively. Note that only residues involved in hydrogen bonding are represented for clarity

Docking calculations of the PHCSBD toward the FP2 binding site revealed they are favourably accommodated within the binding cavity. Both derivatives were preferred to the cocrystallized ligand, a well-known cysteine protease inhibitor called E64 (Table 5). FP2 demonstrated a greater affinity for BepBeH (−10.02 ± 0.78 kcal/mol) than BepINH (−8.11 ± 0.49 kcal/mol) suggesting that hydrophobic energy is the main force driving the protein–ligand interaction. In other words, FP2 had stronger contact with BepBeH than BepINH because the presence of the nitrogen atom in BepINH pyridinyl ring was sufficient to polarize the moiety, which probably resulted in the distortion of the hydrophobic force and subsequently weakened the FP2-BepINH complexation unlike in BepBeH that has phenyl ring in that same position. The presence of multiple aromatic rings in the study compounds ready to make hydrophobic bonding could explain why FP2 preferred interactions with both derivatives to E64 which has zero aromatic ring. The results of Rosenthal and colleagues on the cysteine protease activity of peptidyl fluoromethyl ketones align with our hypothesis since according to their findings, the most potent candidates that inhibited FP2 at subnano- and low-nano-molar concentrations possess more than one aromatic ring [36, 37]. Binding poses of the PHCSBD retrieved from the highest populated clusters revealed the unique docking conformations adopted by both compounds within the FP2 binding site. Interactions with the protein’s Gly83 carboxylate backbone appeared significant as both compounds made polar contact with it (Fig. 2). BepINH made two more hydrogen bonds with Cys42 and Asn81 while BepBeH had only one more with Leu172, however, BepBeH recorded higher binding affinity and this further support the idea that ligand binding to this studied FP2 binding site is majorly determined by hydrophobic force.

**Table 5:** Docking of PHCSBD into FP2 active site

Compound	*K*i (μM)	Binding free energy ligand (kcal/mol)	Ligand (kcal/mol) efficiency
BepBeH	0.93 ± 0.61	−10.02 ± 0.78	0.27 ± 0.09
BepINH	1.14 ± 0.81	−8.11 ± 0.49	0.27 ± 0.09
E64	711.70 ± 0.75	−4.29 ± 0.44	0.23 ± 0.10

E64 = reference (cocrystallized) ligand, *K*_i_ is the theoretical inhibition constant and like binding energy, lower values indicate more favorable interaction. Ligand efficiency is a concept that expresses the sensitivity of binding affinity to an increase in molecular size. The higher the ligand efficiency the better the molecule is as a drug-lead [13]

## Conclusion

Multi-functional compounds have shown amazing biological activities including anti-malarial effect and *Plasmodium* falcipain-2 (FP-2) is an essential validated target in anti-malarial drug discovery. In the present study, new pyrazole-hydrazine coupled Schiff base derivatives were assayed for anti-*Plasmodium* activity in vivo and theoretically docked into the binding site of FP-2. The derivatives significantly reduced parasitaemia count (0.76 ± 1.11 and 0.79 ± 1.19) at day 3 post-treatment and suppressed parasitaemia to levels comparable to ACT-Lonart. In addition, the derivatives showed a good haematological profile. Molecular docking analysis revealed the compounds bound stronger with FP-2 than a known cocrystallized inhibitor and made important interactions with FP-2 binding site residues.

## Supplementary Information

**Additional file 1: Figure S1.** Parasitaemia suppression (%) in prophylactic study. A1: BePINH; A2: BePBeH. **Figure S2.** Parasitaemia suppression (%) in curative study. B1: BePINH; B2: BePBeH.

## Data Availability

All the data related to this work are contained here and in the supporting information.

## Abbreviations

ACT: Artemisinin-based combination therapy
BePINH: (Z)-3-Methyl-1-phenyl-4-(2-phenylhydrazono)-1H-pyrazol-5(4H)-one.
BePBeH: (E)-4-((2-benzylhydrazono) (phenyl)methyl)-3-methyl-1-phenyl-1H-pyrazol-5-ol.
LD: Lethal dose
MOE: Molecular operating environment
Pdb: Protein databank
PPS: Percentage parasitaemia suppression
PHCSBD: Pyrazole-hydrazine coupled Schiff-base derivatives
WHO: World Health Organization
