# Supplementary material for: Evaluation of anti-malarial potency of new pyrazole-hydrazine coupled to Schiff base derivatives
Source: Malar J. 2022 Aug 22;21:243. doi: 10.1186/s12936-022-04266-8 (PMC9396901; doi:10.1186/s12936-022-04266-8)
Supplement: Supplementary file 1 — Additional file 1: Figure S1. Parasitaemia suppression (%) in prophylactic study. A1: BePINH; A2: BePBeH. Figure S2. Parasitaemia suppression (%) in curative study. B1: BePINH; B2: BePBeH. [file 12936_2022_4266_MOESM1_ESM.docx]

**Evaluation of Antimalaria Potency of New Pyrazole-Hydrazine Coupled Schiff Base Derivatives**

Ibezim A.^1^, Ofokansi MN^2^, Ndukwe X^1^, Chiama SC^1^, Obi BC^2^, Isiogugu ON^2^, Peter E Ikechukwu^2^, Onwuka M Akachukwu^2^, Stella A Ihim^4^, Asegbeloyin JN^3^, Nwodo NJ^1^

^1^ Department of Pharmaceutical and Medicinal Chemistry, University of Nigeria, Nsukka

^2^ Department of Pharmacology and Toxicology, University of Nigeria, Nsukka

^3^ Department of Pure and Industrial Chemistry, University of Nigeria, Nsukka

4 Department of science laboratory, University of Nigeria, Nsukka

*For correspondence:

[martha.ofokansi@unn.edu.ng](mailto:martha.ofokansi@unn.edu.ng), +2348037794874


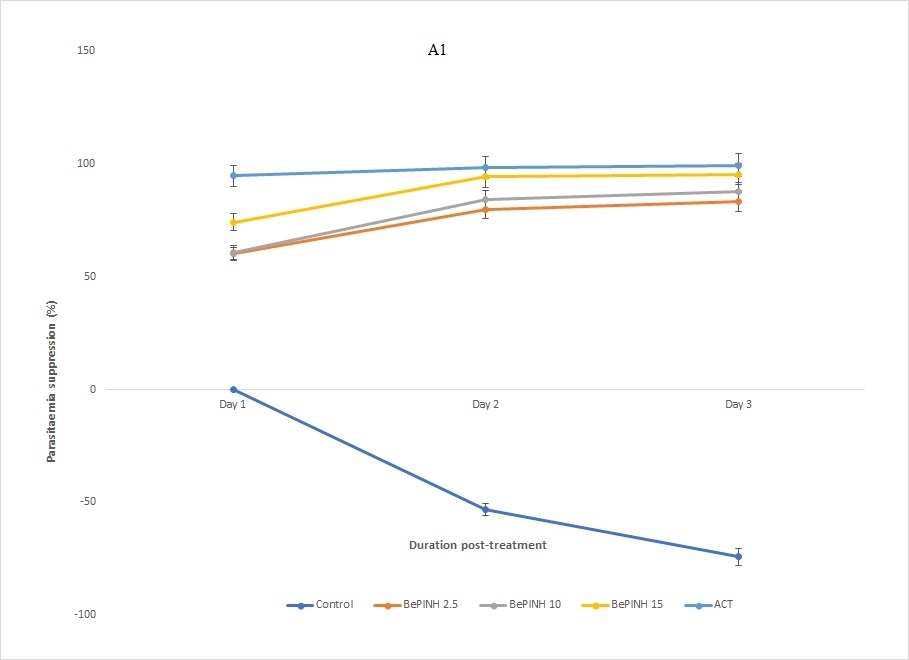


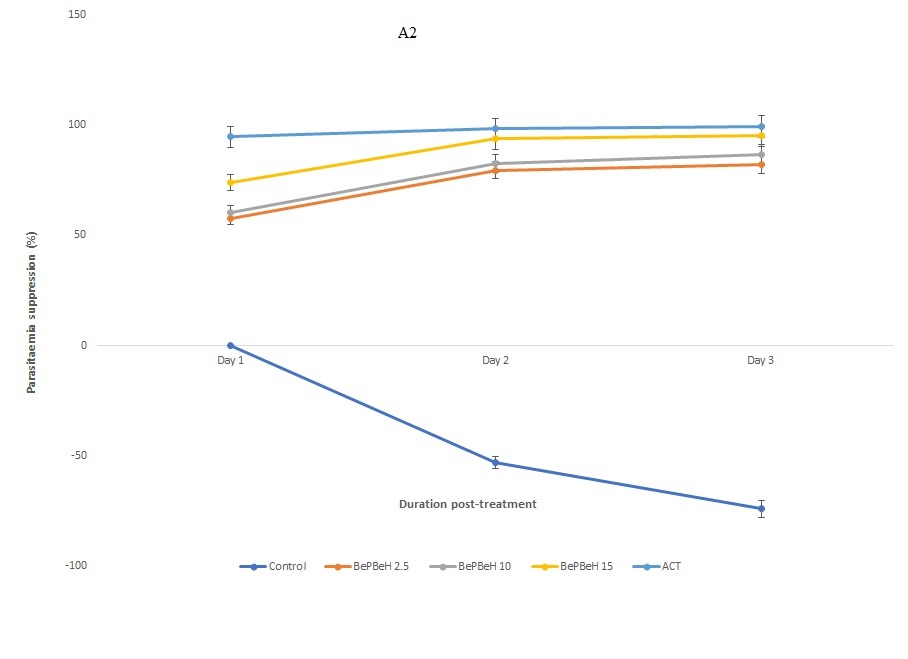


Figure S1. Parasitaemia suppression (%) in prophylactic study. A1: BePINH; A2: BePBeH.


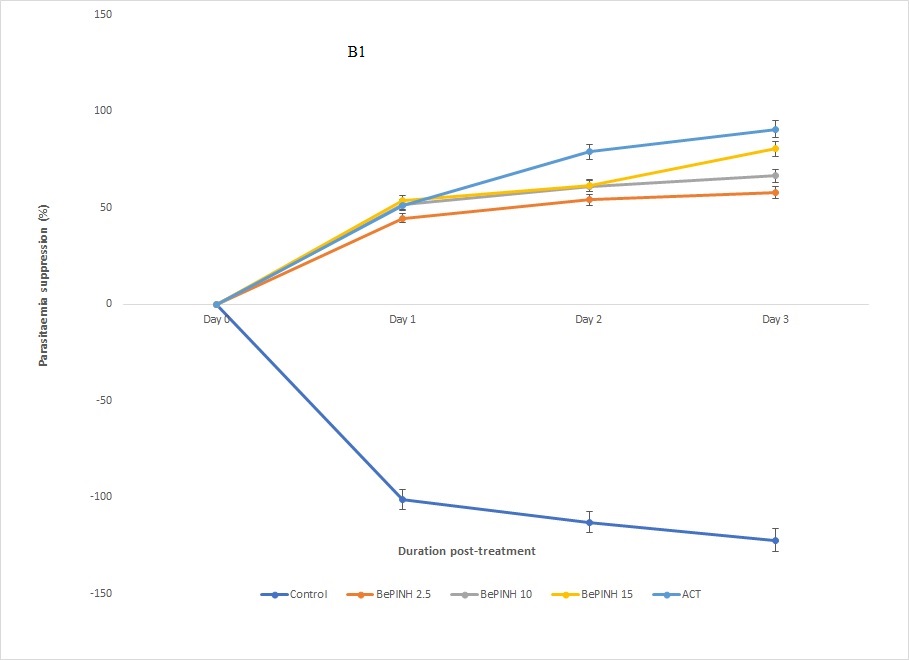


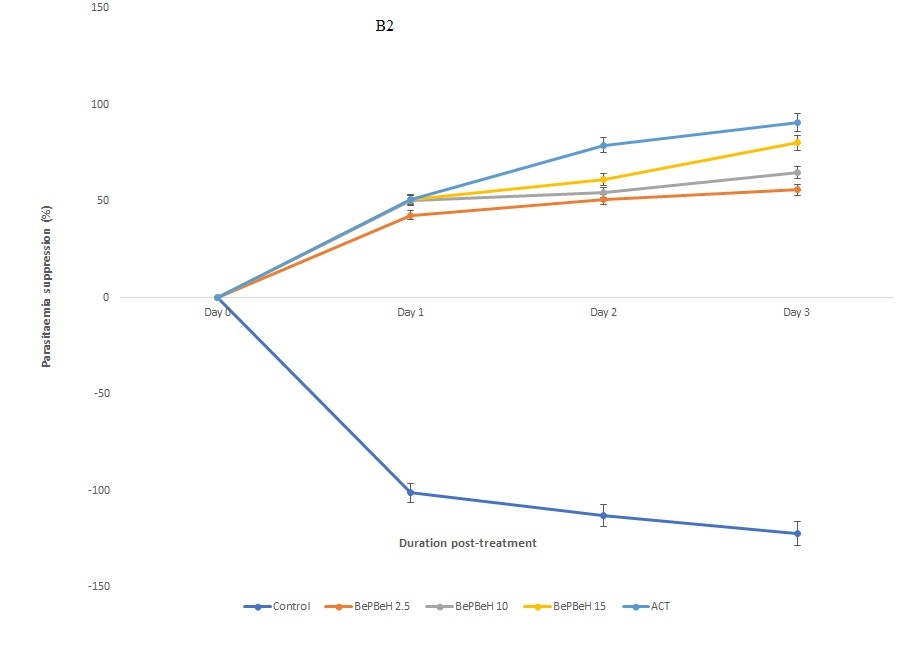


Figure S2. Parasitaemia suppression (%) in curative study. B1: BePINH; B2: BePBeH.
